# Health information and COVID-19 vaccination: Beliefs and attitudes among Japanese university students

**DOI:** 10.1371/journal.pone.0277435

**Published:** 2022-11-09

**Authors:** Masahiko Sakamoto, Ryohei Ishizuka, Chie Ozawa, Yoshiharu Fukuda

**Affiliations:** Teikyo University Graduate School of Public Health, Itabashi, Tokyo, Japan; Universidad Catolica del Norte, CHILE

## Abstract

Coronavirus disease 2019 (COVID-19) vaccination of adolescent and young adults is important for infection control. Factors influencing vaccination behavior in this age group include the source of information about the vaccine and social norms. However, there are few studies on factors influencing vaccination behavior among Japanese university students. This study aimed to assess beliefs, attitudes, and sources of information about the COVID-19 vaccine among university students in Japan. A cross-sectional online questionnaire survey was conducted among students in Teikyo University, Japan in September, 2021. The survey was designed to collect demographic information, vaccination status, attitudes, beliefs, and anxiety about the vaccine, sources of information, and whether or not the participant watched an educational movie. The factors associated with beliefs and attitudes were analyzed using logistic and linear regression. A total of 4,062 valid questionnaires were retrieved. The positive vaccine behavior group included 3,725 students (91.7%). The most common source of information on the COVID-19 vaccine was TV/radio (75.0%), and the most common Social networking service (SNS) for COVID-19 information was Twitter (31.1%). Approximately 85% students believed in the efficacy of vaccination. Positive attitude was associated with female sex and watching an educational movie by the university. Concern about the vaccine was positively associated with information from acquaintances or Instagram, and negatively associated with information from Twitter, and watching the educational movie. The majority of students had positive beliefs and attitudes toward COVID-19 vaccination, and social media and educational movies produced by the university had a large influence on their attitude toward vaccination behavior. On the contrary, some students were anxious about vaccination. Promotion of educational movies on social media by the universities is an effective way to encourage vaccination among young people.

## Introduction

Coronavirus disease 2019 (COVID-19) is a respiratory infectious disease caused by severe acute respiratory syndrome coronavirus 2 (SARS-CoV-2). COVID-19 has caused a pandemic with serious health, economic, and social consequences [1]. Physical distancing, wearing face masks, and importantly, COVID-19 vaccination have been encouraged as preventive measures to control the pandemic [2]. Young adults, including university students, are usually healthy and active. Therefore, they carry a risk of spreading COVID-19 to others [3]. In order to control infection, it is necessary to promote vaccination of young people.

Two COVID-19 vaccines were initially available in Japan: the BNT162b2 vaccine (Pfizer-BioNTech) and mRNA-1273 vaccine (Moderna). The BNT162b2 vaccine was administered mainly to the elderly and health care workers and the mRNA-1273 vaccine to young people. Occupational vaccination programs were recommended to promote vaccination among members of companies and universities.

One of the major challenges in immunization is vaccine hesitancy, which is defined as hesitation or refusal to vaccinate despite the availability of vaccination services [4]. Various factors, including doubt about vaccine’s safety and efficacy, inconvenience (difficulty in accessing the vaccines), and complacency (low risk perception of the disease), that can affect vaccine hesitancy have been identified [4].

Japan is one of the countries where confidence in vaccines is the lowest worldwide [5], and low COVID-19 vaccination rates among young adults have become concerning. Sallam et al. reported that the COVID-19 vaccine acceptance rate is only 47% in Japan [6]. Further, a study has reported that young people in Japan tend to be more unsure and reluctant about receiving the COVID-19 vaccine [7].

In an online survey of 168 US medical students, immediately after COVID-19 vaccine approval, 23% of students were unwilling to be vaccinated [8]. However, many young people had positive attitudes toward vaccination before vaccine approval; in a 2020 survey of 237 US college students, 92% had a positive attitude toward COVID-19 vaccination [9]. In Japan, vaccination coverage among adults in their 20s was nearly 80% at the end of January 2022 [10].

Many factors influence attitudes toward COVID-19 vaccination. In particular, social media, the primary means of information dissemination today, may be a major factor affecting young people [11]. It has been reported that the dissemination of misinformation about the COVID-19 vaccine through social media was associated with lower COVID-19 vaccination rates [12].

In Japan, an effort was made by the government to encourage people to take the COVID-19 vaccine. Social norms are considered to be a strong predictor of health behavior. Dube noted that the vaccine acceptance rate increases when people around unvaccinated people get vaccinated or when their children get vaccinated [13]. In other words, treating vaccination as a social norm may promote acceptance of the vaccine; this is also true for young people. Social norms are reported to be positively correlated with higher COVID-19 vaccination rates among young people [14].

In Japan, majority of young people go to universities; therefore, it is important to target undergraduate students to understand young people’s attitudes toward vaccination. Although several previous studies have reported on factors affecting vaccination behavior among undergraduate students, most studies have focused on medical students [2, 15] and not on the general university students.

There are few studies on factors affecting attitudes toward vaccination among young people in Japan. Teikyo University Hachioji Campus is one of the largest campuses in Japan with approximately 14,000 students, and as a general university, its student population can be considered more generalizable to Japanese undergraduate students. Analyzing these factors may help us develop strategies that may increase positive attitudes among young people.

Previous studies that have examined factors affecting the influenza vaccination using the health belief models have shown that positive attitude towards vaccination increases when accurate information about individual risk, disease severity, and vaccination efficacy is provided by a responsible source of information [16]. Therefore, this study aimed to analyze factors that influence attitudes toward vaccination among Japanese university students, focusing on the sources of health information used by them. We explored the factors by examining beliefs, attitudes, concerns, and sources of information about COVID-19 vaccination based on a questionnaire survey of university students at Teikyo University, Hachioji campus.

## Methods

### 1. Design and study participants

Teikyo University is a general university with 10 faculties and 4 campuses. The university conducted an occupational immunization program for the COVID-19 vaccine for students and staff since July 12, 2021. Preceding this program, the university created a 14-minute-long educational movie to provide information about the COVID-19 vaccine. In this movie, a physician explains about the effectiveness of the COVID-19 vaccine in preventing infection and disease onset and worsening, as well as the possible adverse reactions after vaccination and methods to manage these reactions. The movie was produced by the staff of Teikyo University Graduate School of Public Health and released on the university’s website. The students were encouraged to view the movie via an internal university email. Moreover, the movie was repeatedly broadcasted at the vaccination sites. In September 2021, an online-questionnaire survey on attitudes toward COVID-19 vaccination was administered to university students at the Hachioji campus, Teikyo University. We performed a cross-sectional study using the survey data. We enrolled 14,425 students from the faculties of law, economics, literature, foreign languages, education, and medical technology between September 6 and September 21, 2021. The inclusion criteria were students between the ages of 18 and 30. Students under the age of 18 or over the age of 30 were excluded.

### 2. Data collection

The questionnaire was composed of sociodemographic characteristics, attitudes, beliefs and concerns about the COVID-19 vaccine, sources of information about the vaccine, watching educational movies prepared by the university, and preference for low-evidence information about the vaccine. Sex, age, and grade were included as sociodemographic characteristics. Participants were asked to select information sources from TV/radio, family, acquaintance, website, YouTube, Facebook, Twitter, and Instagram. Attitude toward vaccination was evaluated based on a selection of "already vaccinated," "not vaccinated and plan to be vaccinated," "not vaccinated and hesitant to be vaccinated," or "not vaccinated and do not intend to be vaccinated." Questions on beliefs about the vaccine included "Do you believe it can control transmission to others around you?", "Would you recommend it to your family and friends who have not been vaccinated?", and "Do you believe vaccination is important for getting back to normal life?". Questions regarding concerns about the vaccine included safety concerns due to the short development period and anxiety about adverse reactions after vaccination. Questions regarding information with weak evidence about the vaccine included: "no need for infection control practice after vaccination," "it is better not to vaccinate because the long-term influence is unknown," "the gene is mutated after vaccination," "pregnant women should not be vaccinated," "patients with pre-existing diseases should not be vaccinated," "COVID-19 can be transmitted by vaccination," and "the truth about the vaccine can be known only by watching YouTube."

Data were collected through a self-completed questionnaire using an online university Learning Management System (LMS) through which the university shares information with its students. The survey was voluntary, and students were asked for their consent to participate in the survey prior to completing the questionnaire on the LMS via Google Forms.

### 3. Data analysis

Continuous variables were described as summary statistics (mean and standard error), and categorical variables were described as a frequency and percentage. The attitude toward vaccination was analyzed for sociodemographic characteristics by univariate analysis (χ-square test or Fisher’s exact test). It was classified into positive attitude (completed one or more vaccinations, not yet vaccinated but willing to be vaccinated) and negative attitude (wavering or not planning to be vaccinated). Multiple logistic regression analysis was then performed on attitudes toward vaccination. In the analysis, sex and age were included, and a stepwise procedure was used to select the other variables. The gender and grade ratios of the study population were compared with those all the students at the Hachioji campus to test for selection bias.

In the questions regarding beliefs about the COVID-19 vaccine, the anxiety about adverse reactions and safety concerns (2 questions) were scored on a 5-point Likert scale (5 = "agree" to 1 = "disagree"), and the total score was used as a measure of concern about the COVID-19 vaccine. A t-test was used to evaluate the association between the information sources and concern about the COVID-19 vaccine. Multiple regression analysis was then performed on the concern about the COVID-19 vaccine. A stepwise procedure was used to select variables. Multicollinearity was examined by calculating Variance inflation factor (VIF) for all the variables and was confirmed to be less than 10. A scatter plot of the residuals was created against the predicted values obtained from the model to visually confirm the homoscedasticity of the residuals. Assumption of a normal distribution was confirmed by plotting a QQ plot of residuals. The scatter plots were created in terms of each explanatory variable to confirm the absence of endogeneity.

The statistical significance level was determined as 0.05. JMP Pro 16 (SAS Institute, Cary, North Carolina) was used for data analysis.

Ethical approval was obtained from the Teikyo University Clinical Research and Clinical Trials Review Committee (control code 21–200). With the approval of the Committee, the data from the attitude toward vaccination questionnaire survey conducted via campus e-mail was used secondarily for research purposes. Participants were given a written explanation of the survey, assurance that participation was voluntary and confidential (information provided would be stored in a secure database and used only for research purposes, and results would be unmarked so individuals could not be identified), and consent to the survey was obtained by responding. Participants were also provided with names and phone numbers to contact if further information was needed.

## Results

### 1. Characteristics of the study participants

A total of 4,062 questionnaires were collected (24.1% collection rate). The background characteristics of the study participants and the evaluation results of differences between positive-attitude and negative-attitude groups are summarized in Table 1. Male respondents were 60.4%, and 57% of the respondents watched the educational movie. The rate of vaccination was as follows: 70.9% completed 2 doses, 6.5% completed 1 dose, and 22.6% were unvaccinated. Of the unvaccinated students, 63.2% were "already scheduled or willing to be vaccinated, 24.6% were "wavering," and 12.2% had "no plan to be vaccinated”.

**Table 1:** Background characteristics of the participants and comparison of attitudes toward vaccination (n = 4062).

Characteristic		overall	negative attitude group	positive attitude group	p-value^a^
		n = 4062	n = 337	n = 3725	
Sex	Male	2455(60.4)	223(66.2)	2232(59.9)	0.025*
	Female	1607(39.5)	114(33.8)	1493(40.1)	
Grade	1^st^	1163(28.6)	118(35)	1045(28.1)	0.07
	2^nd^	1120(27.6)	90(26.7)	1030(27.7)	
	3^rd^	885(21.8)	65(19.3)	820(22)	
	4^th^	888(21.9)	63(18.7)	825(22.2)	
	over 5^th^	6(0.15)	1(0.3)	5(0.13)	
choices of low-evidenced information					
	None of the above	3247(79.9)	198(58.8)	3049(81.9)	<0.001*
	no need of infection control practice after vaccination	88(2.2)	3(0.9)	85(2.3)	0.11
	better not to vaccinate because the long-term influence is unknown	168(4.1)	84(24.9)	84(2.3)	<0.001*
	the gene is mutated after vaccination	65(1.6)	22(6.5)	43(1.2)	<0.001*
	pregnant women should not be vaccinated	110(2.7)	12(3.6)	98(2.6)	0.31
	vaccination causes infertility	43(1.1)	13(3.9)	30(0.8)	<0.001*
	patients with pre-existing diseases should not be vaccinated	524(12.9)	73(21.7)	451(12.1)	<0.001*
	COVID-19 can be transmitted by vaccination	19(0.5)	8(2.4)	11(0.3)	<0.001*
	the truth about the vaccine can be known only by watching YouTube	78(1.9)	22(6.5)	56(1.5)	<0.001*
watching educational movie	Yes	2317(57)	47(14)	2270(60.9)	<0.001*
	No	1745(43)	290(86.1)	1455(39.1)	
source of information	healthcare provider	381(9.4)	49(14.5)	332(8.9)	<0.001*
	Father	918(22.6)	78(23.2)	840(22.6)	0.8
	Mother	1444(35.5)	130(38.6)	1314(35.3)	0.23
	other family member	325(8.0)	39(11.6)	286(7.7)	0.012*
	Acquaintance	879(21.6)	79(23.4)	800(21.5)	0.4
	university lecturer	177(4.4)	11(3.3)	166(4.5)	0.3
	newspaper/magazine	815((20.1)	81(24)	734(19.7)	0.057
	TV / Radio	3046(75.0)	240(71.2)	2806(75.3)	0.095
	Website	1516(37.3)	130(38.6)	1386(37.2)	0.62
	YouTube	580(14.3)	79(23.4)	501(13.5)	<0.001*
	Facebook	44(1.1)	3(0.89)	41(1.1)	0.72
	Twitter	1262(31.1)	97(28.8)	1165(31.3)	0.34
	Instagram	170(4.2)	15(4.5)	155(4.2)	0.80

^a^ p-values were calculated using the χ-square test or Fisher exact test.

^a^ p-value indicates a significant difference for the analysis comparing the positive attitude group and negative attitude group.

Positive attitude group: completed at least one vaccination or not yet vaccinated and willing to be vaccinated.

Negative attitude group: not planning to be vaccinated or wavering about vaccination.

Data are expressed as the number (%).

*Statistical significant value(α = 0.05)

### 2. Information source regarding COVID-19 vaccine

**Fig 1:**
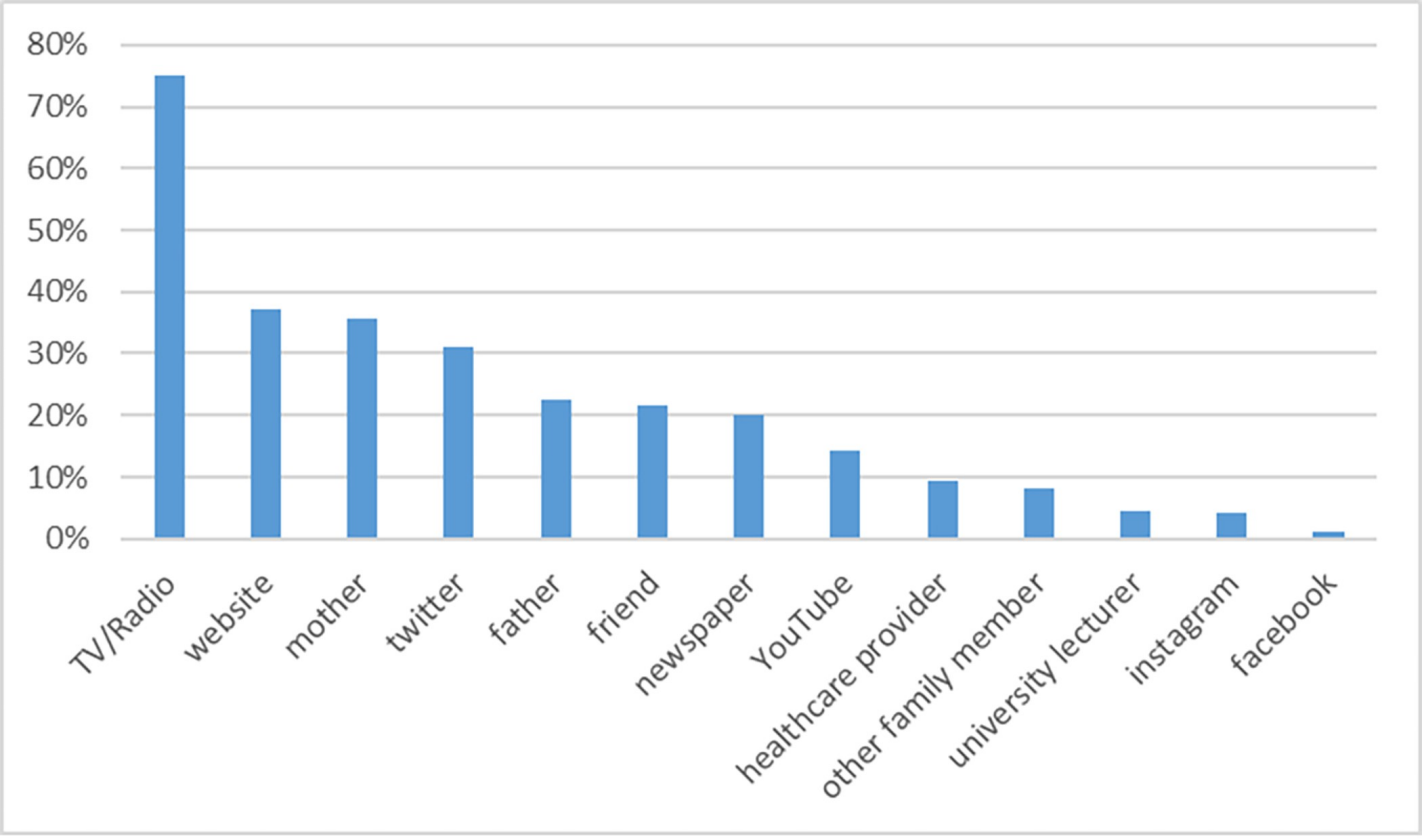
Information sources for the COVID-19 vaccine among university students.

Fig 1 shows the information sources for the COVID-19 vaccine. The most common source was TV/radio (75.0%), followed by websites (37.3%) and mothers (35.5%); among social networking sites, Twitter (31.1%) had the highest percentage, followed by YouTube (14.3%) and Instagram (4.2%).

### 3. Factors associated with vaccination attitudes

Table 1 shows a comparison of vaccination attitudes between the positive group (n = 3725) and the negative group (n = 337). The positive group had significantly more females (40.1% vs. 33.8%, p = 0.025), were more likely to have watched the educational movie (60.9% vs. 14.0%, p<0.01), and were more likely not to choose any of the 8 inaccurate answers regarding the COVID-19 vaccine (81.9% vs. 58.8%, p<0.01). On the other hand, the proportion of students who chose inaccurate choices was higher in the negative group. The proportion of those who chose healthcare providers, family members other than parents, or YouTube as their information source was significantly higher in the negative group (14.5% vs 8.9%, p<0.01; 11.6% vs 7.7%, p = 0.012, and 23.4% vs 13.5%, p<0.01, respectively). The results of multiple logistic regression analysis for positive vaccination attitudes are shown in Table 2. The odds ratio for watching the educational movie was 8.86 (p<0.01), and the odds ratios of collecting information from healthcare professionals and YouTube were 0.58 (p<0.01) and 0.68 (p<0.01), respectively.

**Table 2:** Multiple logistic regression for positive vaccination attitudes.

Variables		Estimated coefficients	SE	OR	95% CI	p-value ^b^
Gender	Female	0.06	0.07	1.12	0.86 to 1.45	0.40
watching the educational movie	Yes	1.09	0.08	8.86	6.40 to 12.3	<0.001*
source of information	Healthcare provider	0.27	0.09	0.58	0.41 to 0.83	<0.001*
	YouTube	0.12	0.08	0.68	0.50 to 0.94	0.012*
choices of low-evidenced information	better not to vaccinate because the long-term influence is unknown	1.09	0.09	0.11	0.08 to 0.16	<0.001*
	patients with pre-existing diseases should not be vaccinated	0.19	0.08	0.68	0.49 to 0.95	0.03*
	the truth about the vaccine can be known only by watching YouTube	0.55	0.18	0.33	0.17 to 0.67	<0.001*

SE, standard error; OR, odds ratio; CI, confidence interval

^b^ p values were calculated using Multiple logistic regression model.

*Statistical significant value(α = 0.05)

### 4. Factors that influenced concern about the COVID-19 vaccine

Those who positively answered "strongly agree" or "agree" to the belief questions, such as "vaccination prevents serious illness" and "reduces transmission to others," were 85.4% and 68%, respectively. In contrast, 48.5% of the participants agreed with the questions "I am worried about safety," and 78.1% agreed with "I am concerned about adverse reactions".

The association between concern about the vaccine and information sources are shown in Table 3. Those who obtained information from their father, mother, acquaintances, and Instagram had higher scores (greater anxiety), while those who gained information from Twitter had lower scores (less anxiety).

**Table 3:** Association between concern about the vaccine and information sources.

information sources	n		Score(average)	SD	p-value ^c^
healthcare provider	No	3681	7.6	1.9	0.46
	Yes	381	7.5	2.1	
Father	No	3144	7.5	1.9	0.049*
	Yes	918	7.7	1.8	
Mother	No	2621	7.5	1.9	0.008*
	Yes	1444	7.7	1.8	
other family member	No	3737	7.6	1.9	0.055
	Yes	325	7.8	1.8	
Acquaintance	No	3183	7.5	1.9	<0.001*
	Yes	879	7.8	1.8	
university lecturer	No	3885	7.6	1.9	0.29
	Yes	177	7.7	2.0	
newspaper/magazine	No	3247	7.6	1.9	0.98
	Yes	815	7.6	2.0	
TV / Radio	No	1016	7.6	2.0	0.87
	Yes	3046	7.6	1.9	
Website	No	2546	7.6	1.9	0.07
	Yes	1516	7.5	2.0	
YouTube	No	3482	7.5	1.9	0.08
	Yes	580	7.7	2.0	
Facebook	No	4018	7.6	1.9	0.41
	Yes	44	7.8	1.9	
Twitter	No	2800	7.6	1.9	0.0039*
	Yes	1262	7.4	1.9	
Instagram	No	3892	7.5	1.9	0.0014*
	Yes	170	8.0	1.8	

SD, Standard Deviation

^c^ P-values were calculated using Student t-test or Welch’s test.

P-values indicate the significant differences between the positive attitude group and the negative attitude group.

*Statistical significant value(α = 0.05)

Table 4 shows the results of the regression analysis using anxiety scores as the objective variable. Concern was higher when the information source was acquaintances or Instagram, when more inaccurate information was selected, or when their attitude towards vaccination was negative. However, their concerns were lower when respondents were males, when their information source was Twitter, or when they had watched educational movies.

**Table 4:** Multiple regression analysis of the predictors of anxiety about COVID-19 vaccine.

Variables		Estimated value	SE	95% CI	p-value†
Male		-0.17	0.03	-0.23 to -0.12	<0.001*
source of information	acquaintance	0.08	0.04	0.01 to 0.15	0.017*
	Twitter	-0.11	0.03	0.04 to 0.17	0.001*
	Instagram	0.23	0.07	0.10 to 0.38	0.002*
number of choices of low-evidenced information		0.48	0.05	0.39 to 0.57	<0.001*
watching the educational movie		-0.09	0.03	-0.15 to -0.04	<0.001*
Negative vaccination attitudes		0.51	0.05	0.40 to 0.62	<0.001*

SE, standard error

† p-values were calculated using Multiple regression analysis

*Statistical significant value(α = 0.05)

## Discussion

In this study, we investigated the beliefs and attitudes regarding COVID-19 vaccination among general university students in Japan, focusing on the sources of health information used by them. The results showed that beliefs and attitudes were positively or negatively related to information sources.

This study showed that television and radio were still the most common sources of information on the COVID-19 vaccine among university students. Although there has been a trend in recent years to focus on information sources other than television, this result suggests that conservative media such as television and radio may still have a significant influence on young people.

On the other hand, a previous study has reported that higher trust in television increases negative attitudes toward the COVID-19 vaccine [7]. Hence, the influence of television on vaccination attitudes should be considered carefully.

In addition, Twitter was selected more frequently than other social networking services (Facebook and Instagram). This suggests that students consider Twitter more important in collecting information on the COVID-19 vaccine. In 2021, Twitter had 260 million daily active users worldwide, with 58 million users in Japan, which is second to the United States [17]. In our study, students who chose Twitter as the source of information showed a lower concern about the COVID-19 vaccine. Thus, it is important to explore the effective use of Twitter. It might be a potentially helpful tool for discussing public health topics, including immunizations, with university students. However, users who prefer scientific news and users who prefer non-scientific news (e.g., conspiracy theories) may belong to separate communities on social media. Therefore, scientific content can reach users who share the same scientific views and is unlikely to reach users who prefer conspiracy theories [18]. Therefore, efforts are required to distribute scientific information comprehensively on social media. Puri et al. suggest that increasing emotional words and images will make messages more persuasive and that fact-checking efforts will help strengthen the quality of content on social media platforms [19].

This study also showed that students were more likely to have negative attitudes toward the COVID-19 vaccine when YouTube was selected as the source of information. Basch et al. examined 87 videos searched on YouTube™ using the keywords "COVID-19 vaccine safety" and "COVID-19 vaccine and children," and found that 65% of them were intended to discourage COVID-19 vaccination [20]. This is consistent with the results of our study. Puri et al. suggest that exposure to these contents, even if only for 5–10 minutes, can negatively affect beliefs toward COVID-19 vaccination [19].

University students were more likely to have negative attitudes toward the vaccine when healthcare professionals were the source of information. This result was contrary to previous studies [9] that showed that recommendations by healthcare professionals encouraged COVID-19 vaccination. This might be explained by the fact that the participants in the study were students of liberal arts, such as law or economics, and had little connection to the medical field. It is possible that medical professionals were not familiar to them, and that the students who consulted with healthcare providers were a highly concerned group. Hence, their major concerns could not have been resolved through consultation alone. With regard to the beliefs of university students toward COVID-19 vaccination, the majority of them held positive beliefs, such as "it prevents people from serious conditions," or "it reduces the possibility of transmission to others." In contrast, approximately 80% held negative beliefs, such as anxiety about adverse reactions and concern about safety. A study of college students in the US reported similar results [9]. Risk perception is considered a predictor of vaccination behavior: the perceived risk of vaccine-preventable disease (VPD) may promote vaccine acceptance, whereas the perceived risk of vaccines may contribute to vaccine refusal [13]. The risks associated with vaccination are known to be perceived as greater than the risks associated with VPD [21]. It should be noted that a large proportion of young people have concerns about adverse reactions and safety of the vaccine.

In the present study, respondents who had watched the educational movie had more positive attitudes toward vaccination. In the movie, the expert explained the effects and adverse reactions of the vaccine in an easy-to-understand manner using illustrations, which may have led to a positive attitude toward vaccination. Previous studies have also shown that educational video messages from experts can increase acceptance of vaccines [22, 23]. The results of this study suggest that educational movies is one of the most effective means of educating undergraduate students about vaccines. While providing information through traditional media and SNS is important, each educational institute should also consider providing information by creating educational movies.

This study has several advantages and disadvantages. The biggest advantage is the large sample size, which allowed researchers to collect information about the COVID-19 vaccine from the students effectively. Moreover, the study’s target population was general university students; therefore, it is not biased toward medical students and the study results are highly generalizable to other young people in Japan. Regarding the limitation of this study, first of all, the collected information was based on self-reports, which are subject to recall bias. Second, it should be noted that the educational movie had also been presented at the vaccination site. This means that those who came to the COVID-19 vaccination site were more likely to have seen the educational movie, and it might not have been a factor contributing to their positive attitude. Third, the low-response rate for the questionnaire (25%) might have led to biased respondents. The study population had a different proportion of gender and grade from the overall student population at the Hachioji campus. Therefore, a possible selection bias cannot be ruled out, as this group may have responded more positively to vaccination. Fourth, the survey included only university students. Higher educational attainment is known to be associated with higher acceptance of the COVID-19 vaccine [24]. Therefore, attitudes toward COVID-19 vaccination in this study might have been more positive than those of average young people, which makes it difficult to apply the findings to all young people. Finally, this study is based on evaluation among student population of a single university. Further research is needed in other regions with young people from different educational backgrounds in order to increase the external validity of the study.

## Conclusion

This study evaluated the sources of information about the COVID-19 vaccine and factors associated with attitudes and beliefs regarding the vaccine among university students. Many undergraduate students expressed concern about the safety of vaccines and had anxiety about the adverse reactions due to COVID-19 vaccination. Addressing causes of their anxiety may lead to positive vaccination behavior. The influence of social media and educational movies on attitudes and beliefs about COVID-19 vaccination was significant, and strategies are required to provide high-quality information. The production of educational movies on social media by universities is an effective way to encourage vaccination among young people.
